# Double duty, shared responsibilities and feedback literacy

**DOI:** 10.1007/s40037-020-00599-9

**Published:** 2020-08-21

**Authors:** David Carless

**Affiliations:** Faculty of Education, University of Hong Kong, Hong Kong, China

Burm et al. designed and implemented an innovation focused on training and deploying medical personnel from one specialism to assess surgical trainees in another specialism [1]. A major finding from the focus group interview data was that learners questioned the credibility of the feedback providers and the constructiveness of the feedback messages. It seemed that learners desired more than an assessment of their competence, they wanted to see how their performance could be improved. For this to transpire, they felt they needed specialist advice from someone who had done similar operations many times before.

As an educational researcher specializing in assessment and feedback, I find much to learn from varied traditions, cultures and perspectives offered by different disciplines. Whilst there are useful generic principles of assessment and feedback, tensions and compromises between generic and discipline-specific features of assessment and feedback practice loom large. There are learning cultures and feedback cultures at the heart of disciplinary practices [2].

A major challenge for assessment practice is that assessment always does double duty [3]: it needs to fulfil multiple functions. Most commonly, and relevant to the contribution of Burm et al., assessment needs to provide a fair appraisal of current achievement whilst at the same time contributing to ongoing development of the individual being assessed.

Feedback information also does double duty [4]. Its functions include: justifying the grade awarded; offering commentary that may be helpful on future tasks; and providing an audit trail for quality assurance purposes. Feedback doing double duty seems highly pertinent to the Burm et al. paper. Learners seemed to want a coach who could support them in advancing their skills but instead they got an assessor who scored their performance. A checklist reinforced this mismatch by itemizing in a procedural way rather than facilitating nuanced feedback interactions. Authentic feedback in medical education might, for example, profitably involve physical demonstration of manual procedures [5].

Feedback exchanges often fail as communication because participants are on different wavelengths. Double duty confounds the difficulties of shared interpretations. Communication and negotiation of goals can minimize misunderstandings and conflicting agendas but need those elusive elements of time, space and longer-term relationships.

The challenges of communication reinforce the social and relational elements of feedback processes. In the section ‘Fostering the feedback alliance’, Burm et al. raise some important issues around relationships and trust. The learners seemed most to crave, ‘competence trust’ [6, 7], a perception that the interlocutor has the capability to provide useful and meaningful feedback that can help them improve their performance.

The concept of the feedback alliance reinforces a need for partnership in feedback processes. Rather than using the term ‘alliance’, educational researchers have talked about ‘shared responsibilities’ in feedback processes [8, 9]. Teachers are responsible for designing opportunities for learners to take action in response to feedback information, whereas learners carry responsibilities to engage with and use feedback.

One of the starting points for Burm et al. was that faculty generally lack assessment expertise. The same can be said for capacities in managing feedback processes. These lacunae are not surprising, faculty have a lot on their plate: updating disciplinary knowledge; obtaining competitive research grants; generating world-class research outputs; teaching in rapidly evolving circumstances; fulfilling administrative duties; and so on. To what extent does the development of assessment and feedback expertise carry weight amidst a multitude of demands? Perhaps it should. After all, assessment drives student learning, and effective feedback processes are one of the most promising ways of improving performance.

The concept of feedback literacy carries potential to contribute to the further development of feedback processes. Student feedback literacy represents the understandings, capacities and dispositions to process and use feedback [10]. To make the most of feedback opportunities, learners need support from educators in designing feedback in ways which enable uptake and development. Promising strategies include embedding the development of feedback literacy within the curriculum [11] and designs for feedback that makes an impact [12].

Burm et al. are to be commended for their honest and transparent reporting of an assessment innovation. The main implications of this commentary are threefold. Assessment and feedback doing double duty needs to be acknowledged and addressed. Feedback processes can flourish when there are feedback alliances and shared responsibilities between educators and learners. The mutual development of educator and learner feedback literacy holds promise in maximizing positive impacts from feedback processes.
